# Supplementary figures and images for: Exploring integument transcriptomes, cuticle ultrastructure, and cuticular hydrocarbons profiles in eusocial and solitary bee species displaying heterochronic adult cuticle maturation
Source: PLoS One. 2019 Mar 14;14(3):e0213796. doi: 10.1371/journal.pone.0213796 (PMC6417726; doi:10.1371/journal.pone.0213796)

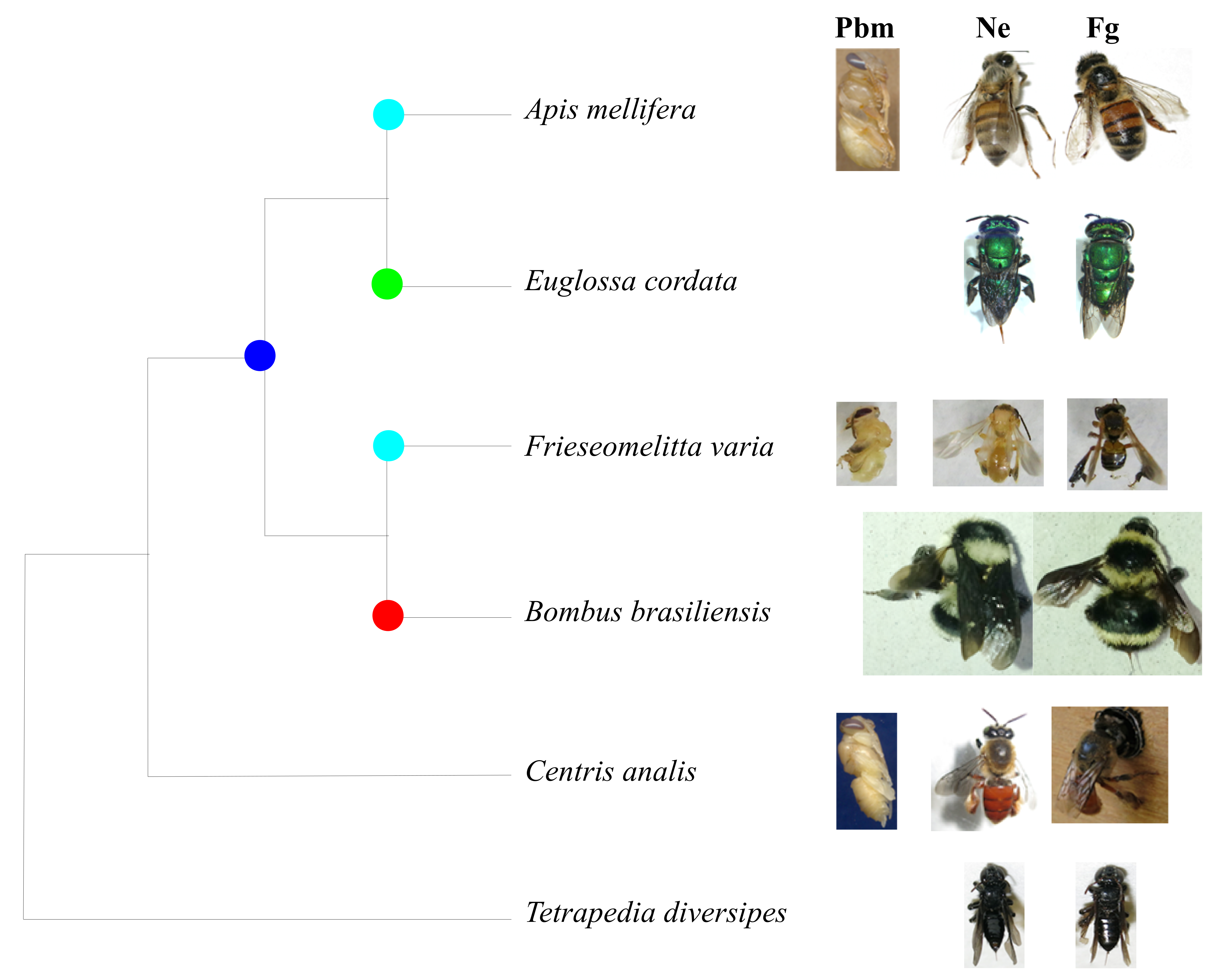

Supplement: S1 Fig — Dark blue: sociality independent of its complexity. Light blue: eusociality. Red: primitively social. Green: facultatively social. Made in LibreOf@ice Draw; based in Kapheim et al. [33]. (TIF) [file pone.0213796.s001.tif]

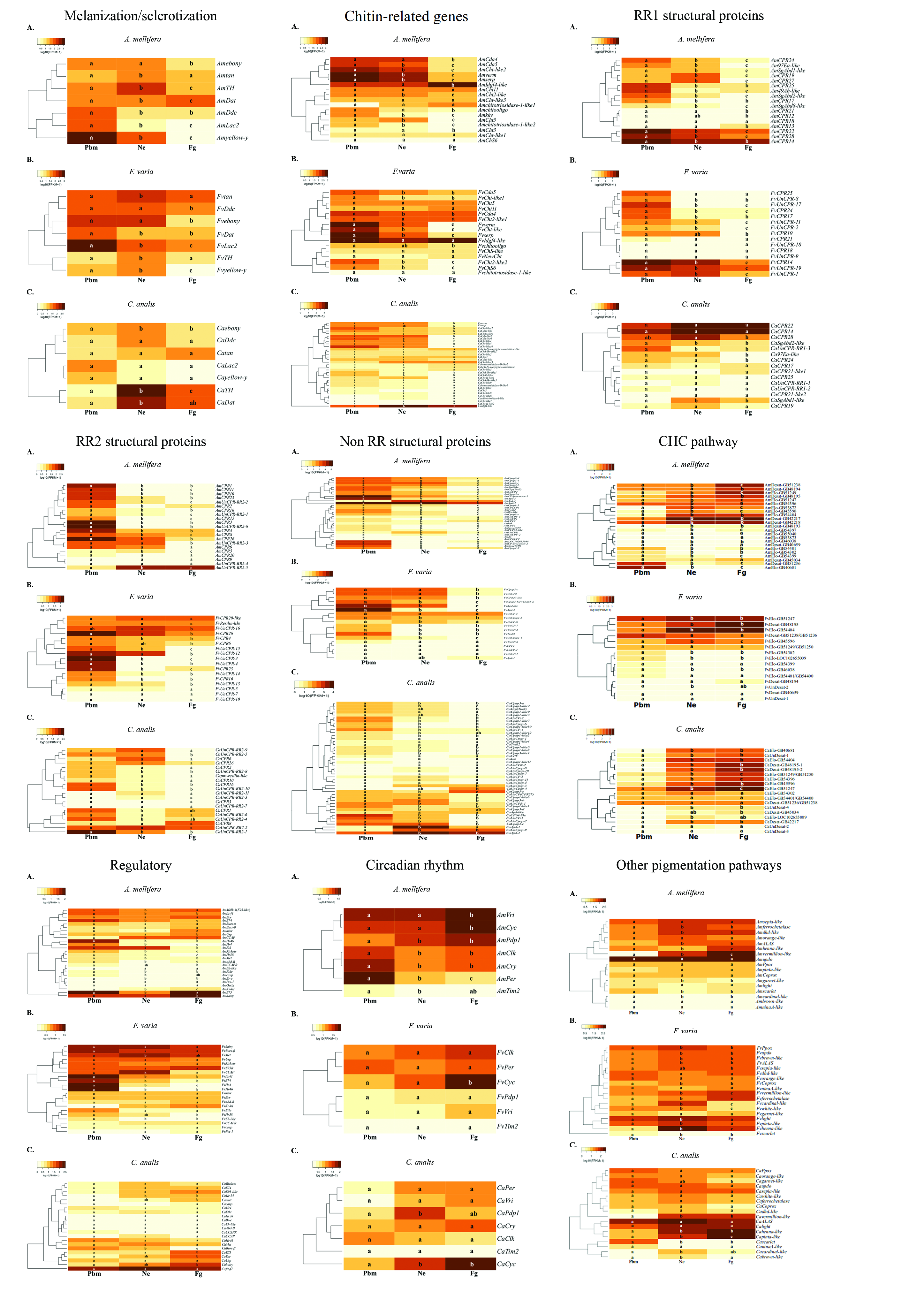

Supplement: S3 Fig — Representative heatmaps of gene expression profiles through the Pbm (pharate-adult), Ne (newly-emerged), and Fg (forager) developmental phases of (A) A. mellifera, (B) F. varia, and (C) C. analis. Genes were grouped according to their potential function in adult cuticle formation and maturation. Different lowercase letters on the heatmaps means statistically significant difference (see Materials and Methods) in the expression levels between the developmental phases of each bee species. (TIF) [file pone.0213796.s003.tif]

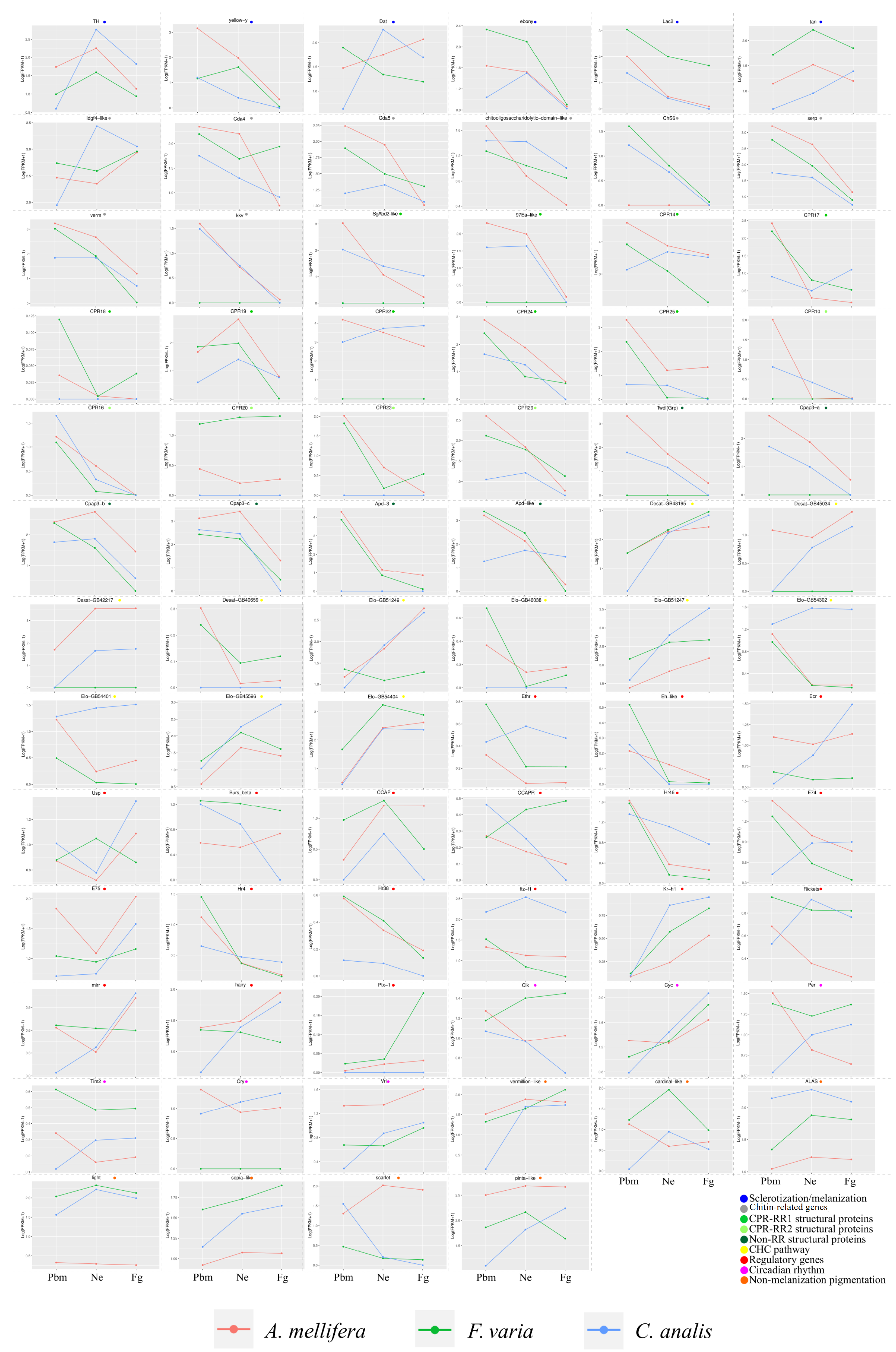

Supplement: S4 Fig — Expression profiles of ebony, tan, Idgf4-like, Cda5, chitooligosaccharidolytic-domain-like, CPR14, CPR17, CPR25, CPR26, Apd-like, Elo-GB54302, Elo-GB54401, Elo-GB45596, Ethr, E74, Hr4, Hr38, FTZ-F1, rickets, Tim2, and ALAS were positively correlated between the eusocial bee species, and negatively or non-correlated with the solitary bee. Expression profiles of CPR18, CPR23, Apd-3, Desat-GB40659, Elo-GB46038, and Ptx-1 were positively correlated between the eusocial species, the basal line in the graphic representations indicating undetected orthologs in C. analis. Pbm: pharate adults, Ne: newly emerged, and Fg: foragers. Color key at the bottom of figure. (TIF) [file pone.0213796.s004.tif]
