# Supplementary material for: Exploring integument transcriptomes, cuticle ultrastructure, and cuticular hydrocarbons profiles in eusocial and solitary bee species displaying heterochronic adult cuticle maturation
Source: PLoS One. 2019 Mar 14;14(3):e0213796. doi: 10.1371/journal.pone.0213796 (PMC6417726; doi:10.1371/journal.pone.0213796)
Supplement: S3 Table — Apis mellifera (A. mel.), Frieseomelitta varia (F. var.), Centris. analis (C. ana.), Bombus brasiliensis (B. bra.), Euglossa cordata (E. cor.), and Tetrapedia diversipes (T. div.). Developmental phases: Pbm (pharate-adult); Ne (newly-emerged, 0h); 24h, 48h, 72h, and 96h after adult emergence; Fg (forager). (DOC) [file pone.0213796.s011.doc]

**S3 Table**. Means and standard deviations of cuticle thickness measurements (μm) in the studied bee species: *Apis mellifera* (A. mel.), *Frieseomelitta varia* (F. var.), *Centris analis* (C. ana.), *Bombus brasiliensis* (B. bra.), *Euglossa cordata* (E. cor.), and *Tetrapedia diversipes* (T. div.). Developmental phases: pharate adults (Pbm); 0h newly-emerged bees (0h Ne); 24h, 48h, 72h and 96 h after emergence; foragers (Fg).

|  | **Pbm** | **Ne (0h)** | **24h** | **48h** | **72h** | **96h** | **Fg** |
| --- | --- | --- | --- | --- | --- | --- | --- |
| **A. mel.** | 7.744±1.392 | 7.445±0.619 | 10.027±2.011 | 9.274±0.519 | 15.169±6.907 | 16.260±3.392 | 16.506± 2.025 |
| **F. var.** | 3.217±0.349 | 3.644±0.111 |  |  |  |  | 3.547±0.326 |
| **C. ana.** | 8.367±1.586 | 24.535±4.251 |  |  |  |  | 26.165±3.515 |
| **B. bra.** |  | 32.950±4.417 |  |  |  |  | 28.460±4.482 |
| **E. cor.** |  | 36.168±3.202 |  |  |  |  | 36.168±8.388 |
| **T. div.** |  | 22.778±11.790 |  |  |  |  | 21.890±1.581 |
